# Dual-Emissive and Color-Tunable Mn-Doped InP/ZnS Quantum Dots via a Growth-Doping Method

**DOI:** 10.1186/s11671-018-2588-0

**Published:** 2018-06-07

**Authors:** Guilin Zhang, Shiliang Mei, Xian Wei, Chang Wei, Wu Yang, Jiatao Zhu, Wanglu Zhang, Ruiqian Guo

**Affiliations:** Engineering Research Center of Advanced Lighting Technology, Ministry of Education; Institute for Electric Light Sources, Fudan University, Shanghai, 200433 China

**Keywords:** Semiconductors, Nanoparticles, InP quantum dots, Mn doping, Dual emission, Color-tunable

## Abstract

In this letter, dual-emissive and color-tunable Mn-doped InP/ZnS quantum dots (Mn:InP/ZnS QDs) with the absolute photoluminescence quantum yield (PL QY) up to 78% were successfully synthesized via a growth-doping method. The dual emission of Mn:InP/ZnS QDs is composed of intrinsic emission and Mn-doped emission, which can be tuned by different Mn/In ratios. With the increase of Mn dopant concentration, the intrinsic emission shows a red shift from 485 to 524 nm. The new class of dual-emissive QDs provides potential for future application in white LED.

## Background

In the past few decades, quantum dots (QDs) have exhibited great potential in biological imaging, fluorescent sensors and optoelectronic devices owing to their unique properties, such as improved thermal and photochemical stability, larger stokes shift and longer photoluminescence (PL) lifetimes [1, 2].

Doped semiconductor QDs have also been widely investigated due to their unique optical properties [3–8]. The PL of QDs can be tailored by doping impurity ions, while their absorption bands remain unchanged. The incorporation of dopants into semiconductor lattices could lead to dual emission consisting of intrinsic emission and doped emission. Compared with the conventional sole-emissive QDs, dual-emissive QDs have some unique advantages in the application of white LED. The dual-emissive QDs have broader PL spectra, which can be easily combined with a blue LED chip to realize white light. As for conventional sole-emissive QDs, two or more kinds of QDs may be required, leading to higher technical difficulties. For years, many efforts have been focused on the cadmium-based QDs due to their unique optical characteristics, but the high toxicity limits their application in many fields. The Mn-doped Zn-Cu-In-S QDs and Mn-doped ZnInS/ZnS QDs have been acting as a new generation of nontoxic dual-emissive QDs. However, because of the low PL QY of no more than 50%, their application potentials are greatly restricted. Recently, InP QDs have been regarded as the most promising candidate to ultimately replace the Cd-based QDs with high toxicity [9–11]. Up till now, a few reports about doped InP QDs have emerged. Peng et al. achieved Cu dopant PL in red and near-infrared window of Cu-doped InP QDs [12], which hinders their application in white LED. The Cu-doped InP core/ZnS barrier/InP quantum well/ZnS shell QDs solve this problem, but the complicated synthetic method makes it difficult to be put into large-scale production [13]. In our previous work, we have studied the synthesis of dual-emissive Ag-doped InP/ZnS QDs [14]. Recently, a report about Ag- and Mn-doped ZnInS/ZnS dual-emissive QDs was published, which can be classified as alloy QDs [15]. The dual emission of the Ag- and Mn-doped ZnInS/ZnS is composed of Ag-doped emission and Mn-doped emission, which is different from the doped InP QDs.

In this letter, dual-emissive Mn:InP/ZnS QDs with the absolute PL QY up to 78% were first synthesized via a growth-doping method. The dual emission of the as-prepared Mn:InP/ZnS QDs is composed of intrinsic emission and Mn-doped emission, which can be tuned by different Mn/In ratios. The new class of dual-emissive QDs provides potential for future application in white LED.

The corresponding PL mechanism was proposed and discussed. The as-obtained QDs were characterized by ultraviolet-visible (UV-vis) spectrophotometry, PL spectroscopy, X-ray diffractometry (XRD), X-ray photoelectron spectroscopy (XPS), high-resolution transmission electron microscopy (HRTEM) and time-resolved fluorescence spectrometry.

## Methods

### Chemicals

Zinc (II) iodide (ZnI_2_, ≥ 98%), tris(dimethylamino)phosphine (P(N(CH_3_)_2_)_3_), 97%), and manganese chloride (MnCl_2_, ≥ 99%) were purchased from Aladdin. Indium (III) chloride (InCl_3_, ≥ 99.995%) was purchased from Acros. 1-Dodecanethiol (DDT, ≥ 98%), 1-octadecene (ODE, ≥ 90%), oleylamine (OLA, 80–90%), and all other solvents were purchased from Sinopharm Chemical Reagent Company. All chemicals were used without further purification.

### Synthesis of Mn:InP/ZnS QDs

Typically, 0.7 mmol InCl_3_, 2.8 mmol ZnI_2_, 6 mL OLA, and 4 mL ODE were loaded in a 50-mL three-neck flask. The mixture was stirred and degassed at 120 °C for an hour and then heated to 220 °C within 10 min under N_2_. 0.25 mL of P(N(CH_3_)_2_)_3_ was rapidly injected into the mixture at 220 °C for the growth of InP core. After 5 min, the solution was heated to 240 °C. Three milliliters DDT, and the MnCl_2_ stock solution obtained by dissolving 0.54 mmol of MnCl_2_ powder into 1 mL ODE and 1 mL OLA at 120 °C, was slowly injected into the InP core crude solution in sequence. After 15 min, the solution was maintained at 200 °C for 5 h and finally cooled to room temperature. As-reacted Mn:InP/ZnS QDs were precipitated twice, using hexane-ethanol extraction by centrifugation (10 min at 7000 rpm). The precipitated particles were dispersed in toluene or hexane.

### Materials Characterizations

All measurements were performed at room temperature. UV–vis and PL spectra were obtained by a Shimadzu UV-3600 ultraviolet spectrophotometer and a Shimadzu RF-5301PC fluorescence spectrophotometer. TEM data were obtained on a JEOL2100F field emission source transmission electro-microscope operating at 200 kV. X-ray diffraction experiments were performed using Bruker D8 Advance. XPS studies were performed on an ESCALAB250Xi X-ray photoelectron spectrometer. PL decay data was obtained on a FLSP920 Steady State and Transient State Fluorescence Spectrometer.

The absolute photoluminescence quantum yield (PL QY, *Φ*_*pl*_) was measured by an integrating sphere on a FLSP920 Steady State and Transient State Fluorescence Spectrometer. This involves the determination of the absorbed photon flux (${q}_p^{abs}$) and the emitted photon flux (${q}_p^{em}$) by a sample (see Eq. (1)) using an integrating sphere setup.

1 $$ {\varPhi}_{pl}=\frac{\int_{\lambda_{em1}}^{\lambda_{em2}}\frac{\Big({I}_x\left({\lambda}_{em}\right)-{I}_b\left({\lambda}_{em}\right)}{s\left({\lambda}_{em}\right)}{\lambda}_{em}d{\lambda}_{em}}{\int_{\lambda_{ex1}}^{\lambda_{ex2}}\frac{\Big({I}_b\left({\lambda}_{ex}\right)-{I}_x\left({\lambda}_{ex}\right)}{s\left({\lambda}_{ex}\right)}{\lambda}_{ex}d{\lambda}_{ex}}=\frac{q_p^{em}}{q_p^{abs}} $$

where *I*_*x*_(*λ*_*em*_)/*s*(*λ*_*em*_) and *I*_*b*_(*λ*_*em*_)/*s*(*λ*_*em*_) represent the counts of the sample emission and blank emission, respectively; *I*_*x*_(*λ*_*ex*_)/*s*(*λ*_*ex*_) and *I*_*b*_(*λ*_*ex*_)/*s*(*λ*_*ex*_) represent the counts of the sample scatter and blank scatter, respectively.

## Results and Discussions

### Crystalline Nanostructures and Composition Measurements

**Fig. 1:**
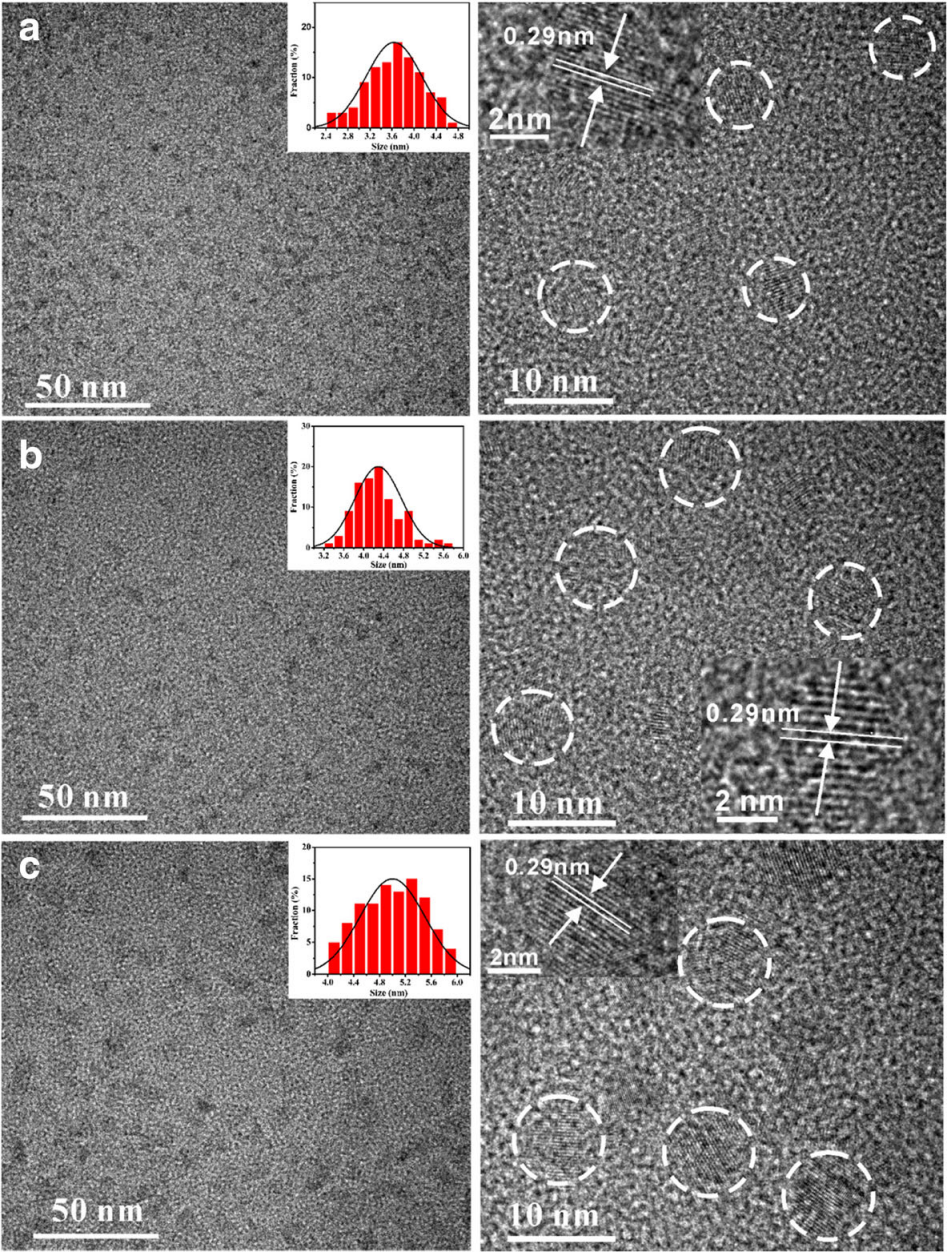
TEM and HRTEM images of **a** InP/ZnS QDs (Mn/In = 0), **b** Mn:InP/ZnS QDs (Mn/In = 0.4), and **c** Mn:InP/ZnS QDs (Mn/In = 0.6). The inset of the HRTEM images corresponds to single QD at high magnification while the scale bar is 2 nm

Figure 1 shows the TEM and HRTEM images of Mn:InP/ZnS QDs with different Mn/In ratios. Particle size distributions (the inset images) reveal the Mn:InP/ZnS QDs with average size of 3.6 nm (Mn/In = 0), 4.3 nm (Mn/In = 0.4), and 5.0 nm (Mn/In = 0.6), respectively. It can be concluded that the size of Mn:InP/ZnS QDs obviously increases with the Mn/In ratio increasing, in accordance with the HRTEM results.

When the halides are adsorbed at the InP surface, either a different binding strength or changing steric effects may lead to systematic variations of the surface-reaction rate constants [9]. In particular, the less voluminous chloride ions may increase surface-reaction rates. In this case, the stock solution of MnCl_2_ is injected into the mixture as manganese raw materials. The chloride ions adsorbed at the InP surface speed up the rates of surface reaction and thus increase the size of the QDs. Higher concentration of the chloride (increased with the Mn/In ratio) leads to bigger size of the Mn:InP/ZnS QDs.

**Fig. 2:**
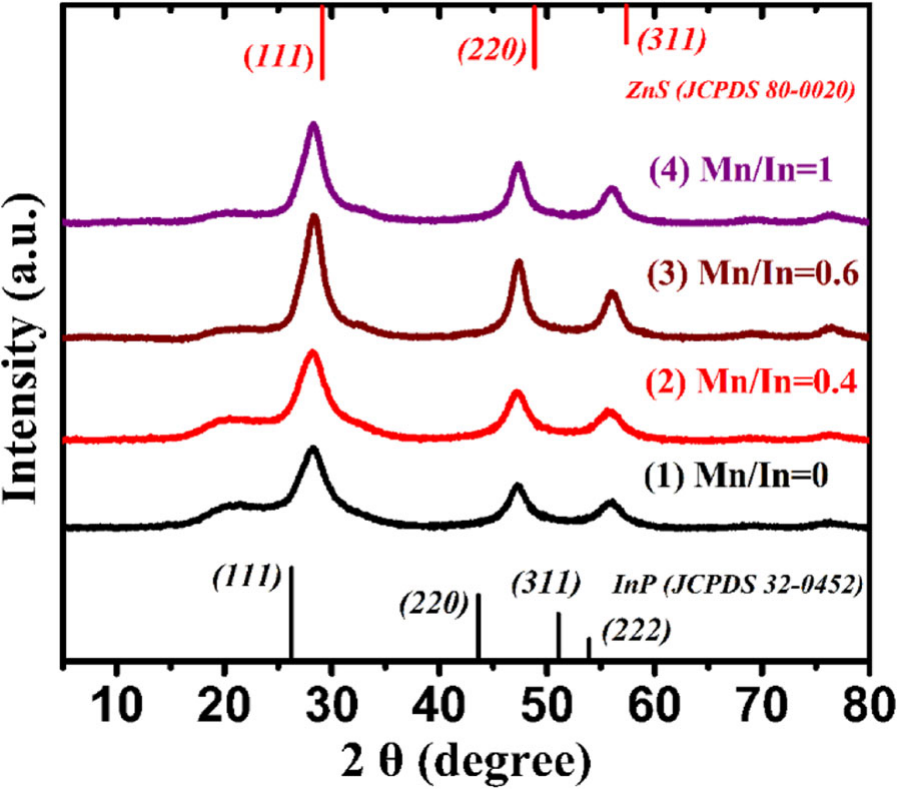
The XRD patterns of Mn:InP/ZnS QDs with different Mn/In ratios

**Fig. 3:**
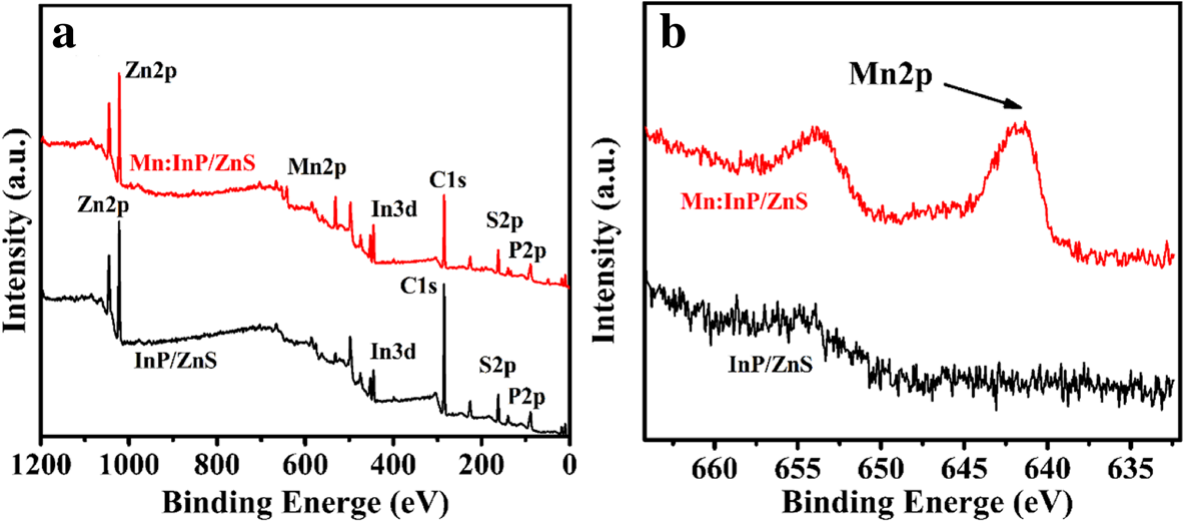
**a** XPS patterns of InP/ZnS and Mn:InP/ZnS QDs. **b** HRXPS patterns of Mn

Figure 2 shows the XRD patterns of Mn:InP/ZnS QDs with different Mn/In ratios. For comparison, the diffraction peaks of bulk ZnS and InP crystals were marked in Fig. 2. The XRD patterns for the Mn:InP/ZnS QDs with three broadened diffraction peaks at 28.3°, 47.3° , and 55.8° under different Mn/In ratios correspond to (111), (220), and (311) facets. The results indicate that all the samples have the same zincblende (cubic) structure, coinciding with the previous reports for the InP/ZnS QDs [16, 17]. Besides, the diffraction peaks are located between the cubic InP and ZnS bulk materials and there are no diffraction peaks of separate ZnS or InP phase, indicating that the ZnS shell is successfully formed on the InP core. It can be concluded that the as-prepared InP/ZnS QDs possess core shell structure and the introduction of Mn ions into InP host will not change its crystal structure. Moreover, the XPS patterns of InP/ZnS and Mn:InP/ZnS QDs are presented in Fig. 3a, respectively. They show the identical peaks, which can be identified as Zn, In, P, and S. However, the peak of Mn2p at binding energy of 642.2 eV in the XPS pattern of Mn:InP/ZnS QDs occurs, as shown in Fig. 3b, indicating the effective introduction of Mn ions into InP host.

Table 1 is about the detailed element content of the Mn:InP/ZnS QDs (Mn/In = 0.4), which presents that the real Mn/In ratio of Mn:InP/ZnS QDs (Mn/In = 0.4) is 1.40. The real content is different from the nominal precursor molar ratio (Mn/In = 0.4), which is probably because part of P and In ions could not participate in the growing process of InP core. Besides, the small size of QDs and the sparse distribution in the solution could also lead to the deviated characterization.

**Table 1:** The detailed element content of Mn:InP/ZnS QDs (Mn/In = 0.4)

Element	Atomic %
C	72.1
Zn	10.51
S	10.55
In	1.58
P	3.05
Mn	2.21

### Optical Characterization of Mn:InP/ZnS QDs

**Fig. 4:**
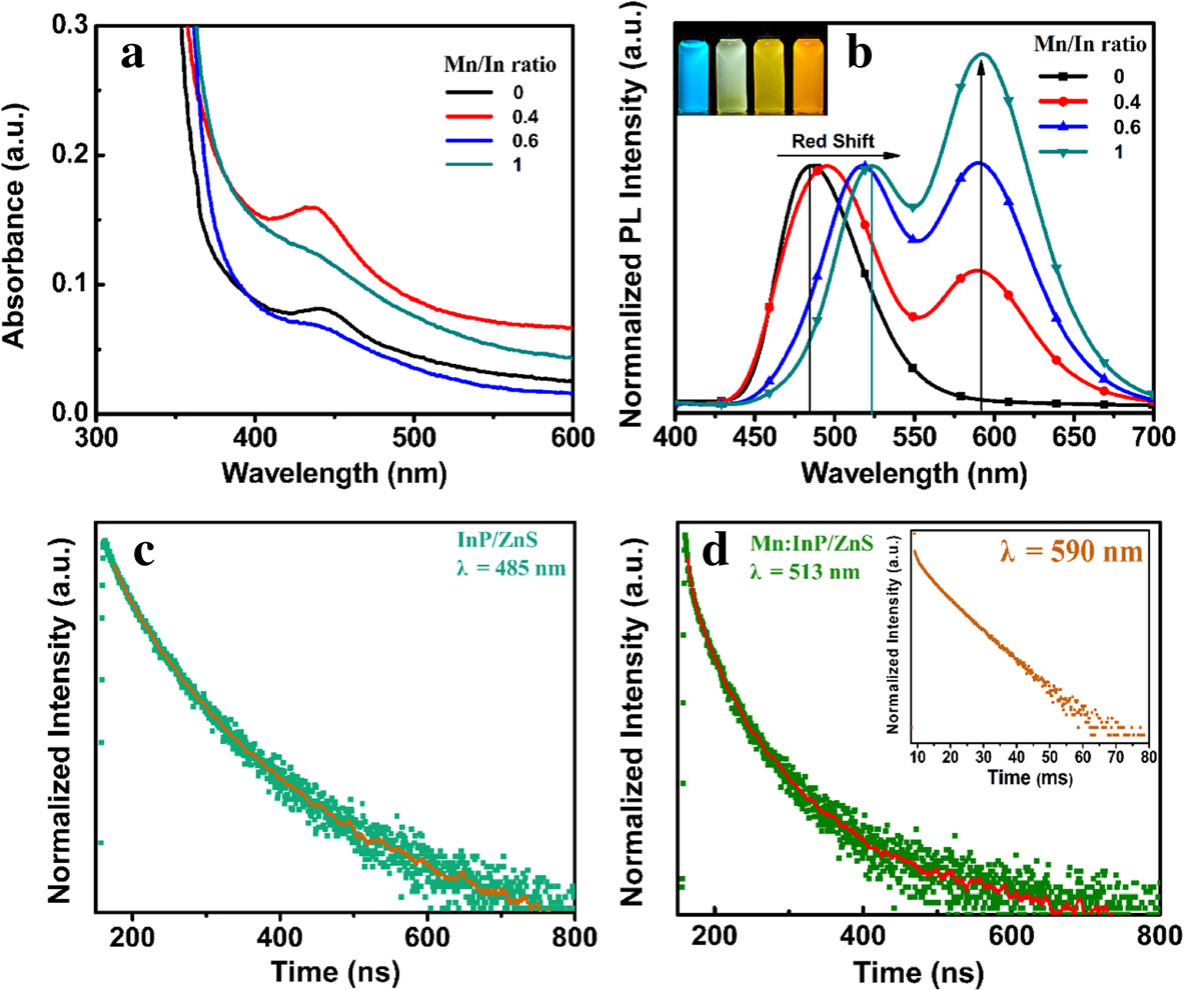
**a** The UV-vis absorption and **b** PL spectra of Mn:InP/ZnS QDs (*λ*_*ex*_ = 360 nm) with different Mn/In ratios. Time-resolved PL decay curves of **c** InP/ZnS QDs with emission wavelength of 485 nm, *λ*_*ex*_ = 360 nm and **d** Mn:InP/ZnS with emission wavelength of 513 and 590 nm, *λ*_*ex*_ = 360 nm (Mn/In = 0.6). The solid lines represent fitting curves

Figure 4a, b represents the UV-vis absorption and PL spectra of Mn:InP/ZnS QDs with different Mn/In ratios, respectively. Figure 4a presents the excitonic absorption peak of Mn:InP/ZnS QDs at 445 nm, and there is no significant change with different Mn/In ratios. When the Mn/In ratio was 1, the excitonic absorption peak became unobvious. The PL peak of InP/ZnS QDs (Mn/In = 0) at 485 nm is assigned to be the intrinsic emission of InP core. For Mn:InP/ZnS QDs, it can be observed that a new peak centered at 590 nm occurs, which is usually perceived as the Mn-doped emission. The emission intensity at 590 nm enhances with the increase of Mn/In ratio, which could be ascribed to the incorporation of more Mn ions into the host lattice to act as recombination centers. Interestingly, with the increase of Mn/In ratio, the intrinsic emission shows a red shift from 485 to 524 nm. This large shift can be explained by the HRTEM results, that is, the higher Mn/In ratio leads to a larger size of Mn:InP/ZnS QDs.

The PL mechanism can be analyzed by the PL decay curves of the InP/ZnS and Mn:InP/ZnS QDs, respectively, as shown in Fig. 4c, d.

The PL decay curves of the intrinsic emission and Mn-doped emission were fitted with triexponential and biexponential functions as follows, respectively. The fitting parameters are given in Table 2.

$$ {\displaystyle \begin{array}{l}{f}_1(t)={a}_1{e}^{-t/{t}_1}+{a}_2{e}^{-t/{t}_2}+{a}_3{e}^{-t/{t}_3}\left({a}_1+{a}_2+{a}_3=1\right)\\ {}{f}_2(t)={a}_1{e}^{-t/{t}_1}+{a}_2{e}^{-t/{t}_2}\left({a}_1+{a}_2=1\right)\end{array}} $$

**Table 2:** Decay times and amplitude constant ratios of PL emission for InP/ZnS (Mn/In = 0) and Mn:InP/ZnS (Mn/In = 0.6) QDs

	*a*_1_	*a*_2_	*a*_3_	*t*_1_	*t*_2_	*t*_3_
InP/ZnS (485 nm)	0.127	0.522	0.351	21.8 ns	60.0 ns	274.6 ns
Mn:InP/ZnS (513 nm)	0.137	0.597	0.266	23.2 ns	63.5 ns	202.7 ns
Mn:InP/ZnS (590 nm)	0.206	0.794	–	2.5 ms	6.0 ms	–

According to Table 2, the PL lifetime (*τ*_*av*_) of InP/ZnS QDs is 217 ns. The PL decay curves of Mn:InP/ZnS QDs (Mn/In = 0.6) were also collected at different emission wavelengths (Fig. 4d and Table 2). When monitored at 513 nm, the resulting *τ*_*av*_ of 141 ns is close to that of undoped QDs since intrinsic emission was well separated from Mn-doped emission. Meanwhile, with monitoring at 590 nm, very long decay behavior with a *τ*_*av*_ of 5.6 ms, characteristics of d-d transition of Mn ion, is observable. As a result, the two emission peaks of Mn:InP/ZnS QDs attributed to the intrinsic emission and Mn-doped emission can be confirmed.

**Fig. 5:**
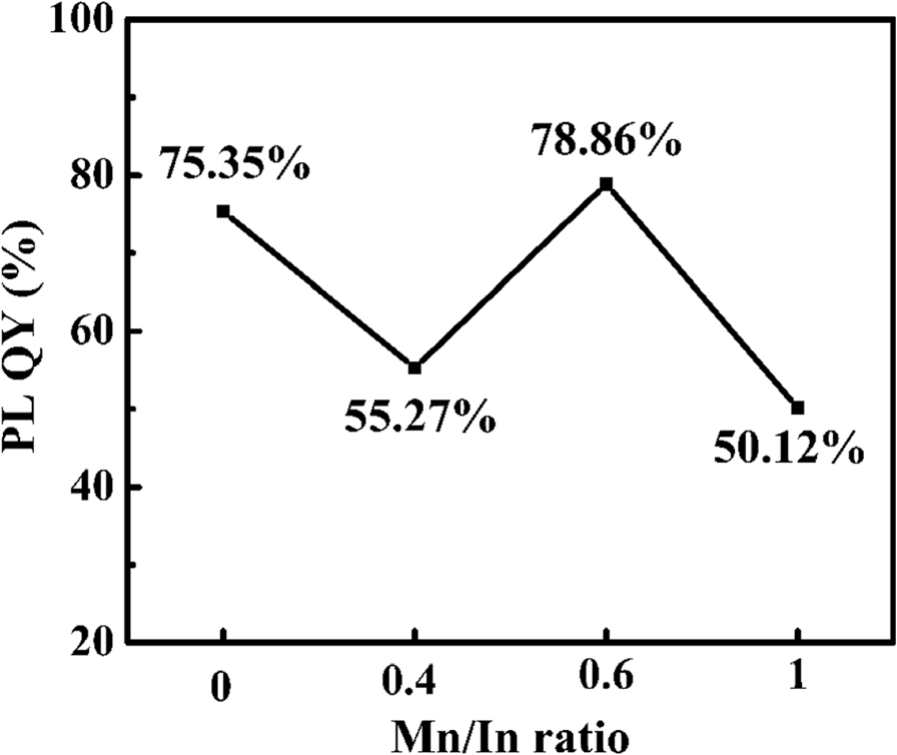
The absolute PL QY of Mn:InP/ZnS QDs with different Mn/In ratios

Figure 5 represents the absolute PL QY of Mn:InP/ZnS QDs with different Mn/In ratios. Generally, the introduction of Mn leads to the decrease of the intrinsic luminescent centers of InP. When the amount of Mn dopant is relatively small, the Mn-doping luminescent centers increased limitedly; however, the luminescent centers of InP decreased greatly. As a result, the whole PL QY was reduced. Meanwhile, when the ratio of Mn/In changes between 0.4 and 0.6, the increasing concentration of Mn has little effect on the decrease of intrinsic luminescence of InP, leading to the PL QY enhancement. And when the ratio of Mn/In reaches 0.6, the PL QY of Mn:InP/ZnS QDs rises to 78.86% due to the increasing luminescent centers of Mn. With the further increase of Mn dopant concentration, the intrinsic luminescence of InP further quenches, and the high doping concentration will also lead to more non-radiative centers, which could reduce the PL QY. Thus, appropriate Mn/In ratio is one of the crucial factors to the PL QY of Mn:InP/ZnS QDs.

### Mechanism Insights of the Dual Emission

**Fig. 6:**
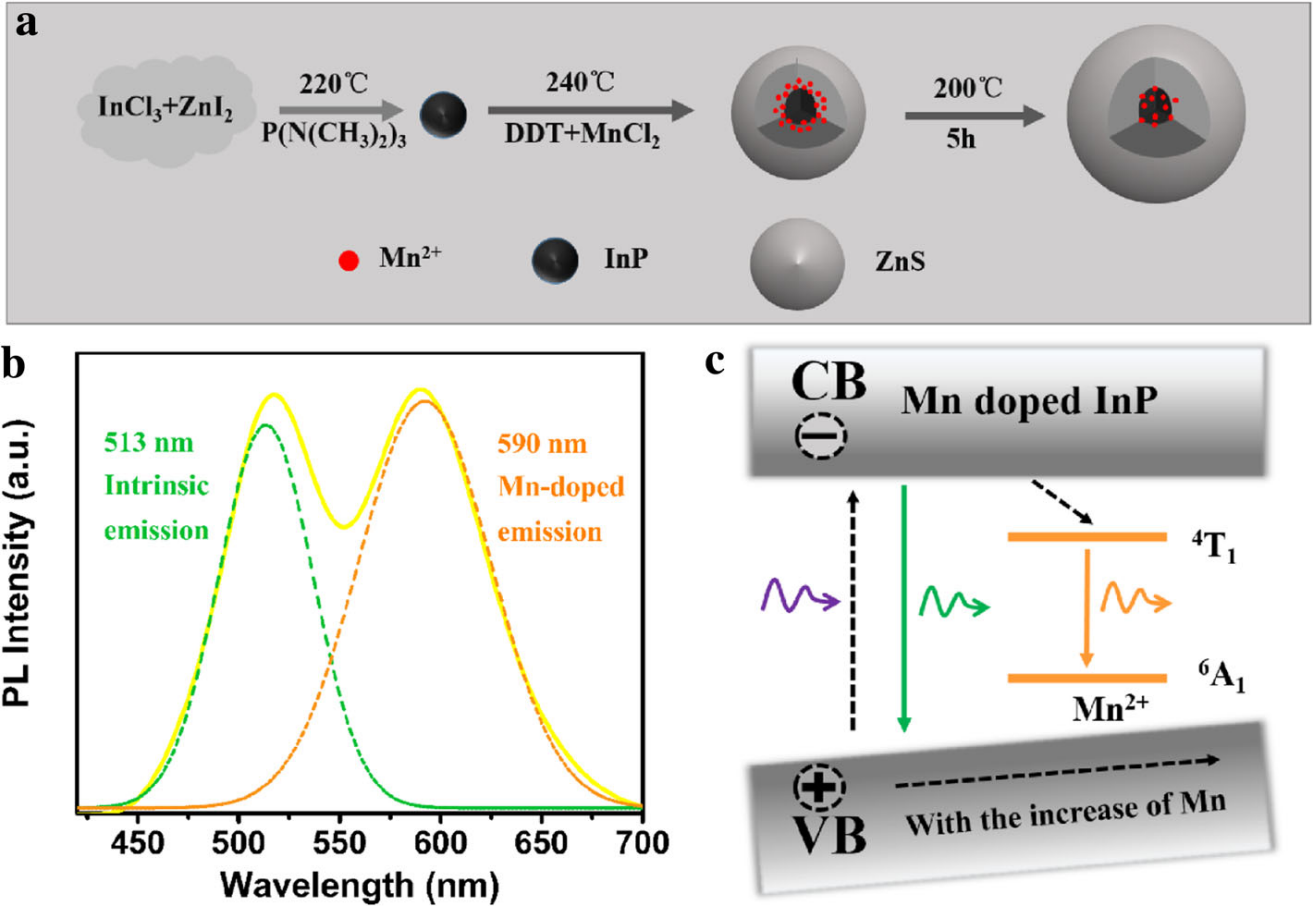
**a** Schematic diagram for the synthetic process of Mn:InP/ZnS QDs. **b** Emission peak fitting result of Mn:InP/ZnS QDs (Mn/In = 0.6) consisting of intrinsic emission and Mn-doped emission. **c** Schematic representation for the recombination mechanism of Mn:InP/ZnS QDs

To further understand the mechanism of growth-doping for dual emission, the synthetic scheme is illustrated in Fig. 6a. The InP core is formed at 220 °C, then the Mn doping process is operated at 240 °C after the injection of DDT. It favors to introduce more Mn ions to the surface of InP core because of the rich anions released from DDT [18–20], which contributes to the growth of nanocrystals with less lattice mismatch, and much symmetric crystal lattices. Emission peak fitting result of Mn:InP/ZnS QDs (Mn/In = 0.6) in Fig. 6b obviously reveals that dual emission contains intrinsic emission and Mn-doped emission. A plausible mechanism schematic for this phenomenon is exhibited in Fig. 6c. The dual emission originates from two different excited states within the QDs, the recombination of the electrons from the conduction band (CB) and holes from the valence band (VB), and the recombination of the electrons from the ^4^T_1_ state and holes from the ^6^A_1_ sate of Mn ion [21, 22]. With the Mn dopant concentration increasing, the bandgap of host becomes narrower, resulting in the red shift of intrinsic emission.

## Conclusions

In summary, dual-emissive and color-tunable Mn:InP/ZnS QDs with the absolute PL QY of 78% were first synthesized via a growth-doping method. The PL spectrum of Mn:InP/ZnS QDs consists of two emission peaks corresponding to intrinsic emission and Mn-doped emission. With the increase of Mn dopant concentration, the intrinsic emission shows a red shift from 485 to 524 nm due to the increasing size of Mn:InP/ZnS QDs. Herein, the new class of dual-emissive QDs provides much potential for future application in white LED.

## Abbreviations

CB: Conduction band
DDT: 1-Dodecanethiol
Fig: Figure
h: Hour
HRTEM: High-resolution transmission electron microscopy
InCl_3_: Indium (III) chloride
LED: Light-emitting diode
min: Minute
Mn:InP/ZnS QDs: Mn-doped InP/ZnS quantum dots
MnCl_2_: Manganese chloride
ODE: 1-Octadecene
OLA: Oleylamine
P(N(CH_3_)_2_)_3_: Tris(dimethylamino)phosphine
PL QY: Photoluminescence quantum yield
PL: Photoluminescence
QDs: Quantum dots
rpm: Revolution per minute
TEM: Transmission electron microscopy
UV-vis: Ultraviolet-visible
VB: Valence band
XPS: X-ray photoelectron spectroscopy
XRD: X-ray diffractometry
ZnI_2_: Zinc (II) iodide
